# Nanoformulation-Based Transdermal Drug Delivery: A Paradigm Shift in Antiparasitic Therapy for Zoonotic Diseases

**DOI:** 10.3390/pharmaceutics17091216

**Published:** 2025-09-18

**Authors:** Yuan Zhao, Ruoxuan Xiu, Chengxiang Wang, Junqi Wang, Dawei Guo, Wanhe Luo, Shanxiang Jiang, Zhiyi Ge, Xiuge Gao

**Affiliations:** 1 Joint International Research Laboratory of Animal Health and Food Safety, College of Veterinary Medicine, Nanjing Agricultural University, 1 Weigang, Nanjing 210095, China; 2023807142@stu.njau.edu.cn (Y.Z.); 2024807139@stu.njau.edu.cn (R.X.); cxw55@stu.njau.edu.cn (C.W.); 2021207003@stu.njau.edu.cn (J.W.); gdawei0123@njau.edu.cn (D.G.); jiangsx66@njau.edu.cn (S.J.); 2 Center for Veterinary Drug Research and Evaluation, Laboratory of Veterinary Pharmacology and Toxicology, College of Veterinary Medicine, Nanjing Agricultural University, 1 Weigang, Nanjing 210095, China; 3 College of Animal Science and Technology, Tarim University, Alar 843300, China; luowanhe0728@163.com; 4 Engineering Laboratory of Tarim Animal Diseases Diagnosis and Control, Xinjiang Production & Construction Corps, Alaer 843300, China

**Keywords:** nanoparticles, transdermal drug delivery, antiparasitic drug, leishmaniasis, malaria, one health, zoonotic diseases

## Abstract

Nanoparticle-based transdermal drug delivery systems (TDDS) have emerged as a revolutionary approach for antiparasitic therapy, addressing key challenges such as poor bioavailability, systemic toxicity, and drug resistance. This review highlights the advancements in nanotechnology-driven TDDS for combating zoonotic parasitic diseases, including leishmaniasis, malaria, and infections treated by broad-spectrum drugs like ivermectin and albendazole. By leveraging nanocarriers such as liposomes, nanoemulsions, and microneedles, which enhance skin permeation, enable controlled drug release, and improve targeting specificity. For instance, deformable transfersomes and ethosomes achieve high transdermal efficiency without chemical adjuvants, while microneedle arrays physically bypass the stratum corneum for precise delivery. Furthermore, sustained-release hydrogels and stimuli-responsive nanoparticles optimize therapeutic efficacy and reduce adverse effects. Despite promising results, clinical translation faces challenges in manufacturing scalability, long-term safety, and accessibility in resource-limited settings. Future directions include bioinspired nanocarriers, artificial intelligence (AI)-driven design, and integration with global health initiatives like “One Health”, all aimed at ensuring equitable implementation. This review highlights the transformative potential of nanotechnology in achieving sustainable antiparasitic solutions for zoonotic diseases.

## 1. Introduction

**Figure 1:**
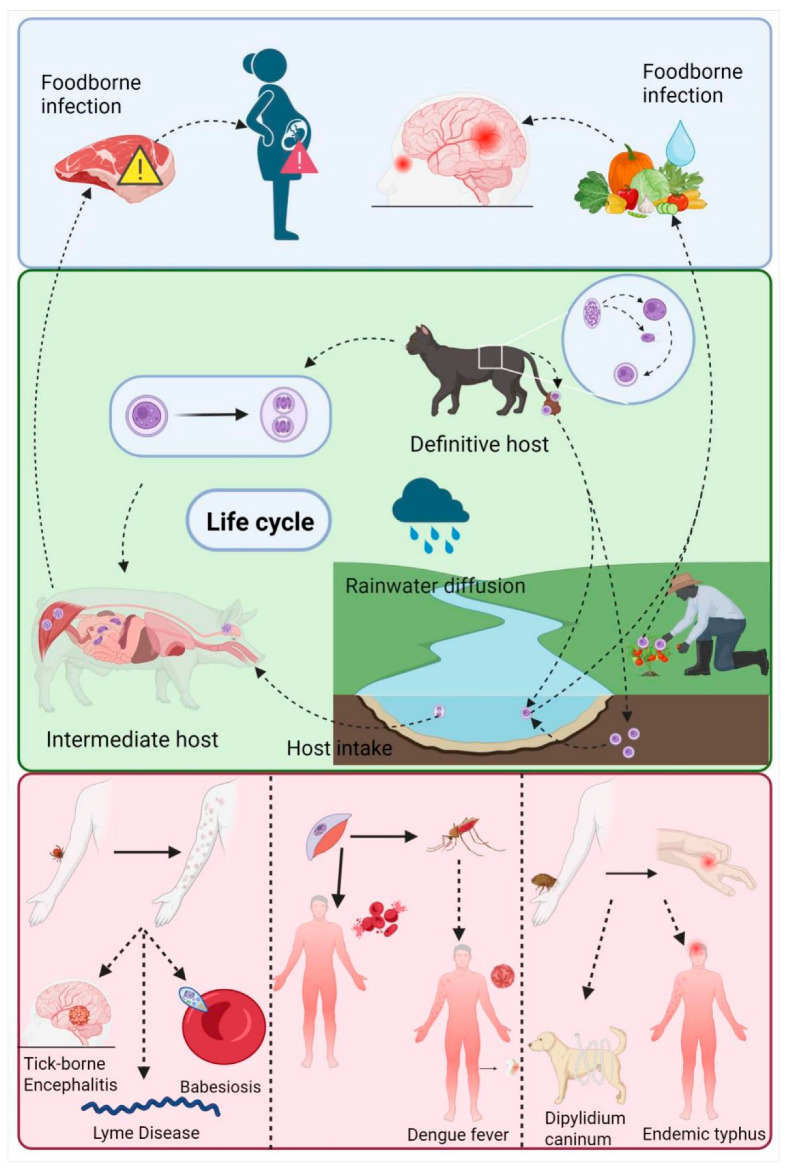
Zoonotic Parasitic Diseases under the One Health Framework (Take Toxoplasma gondii as an example). This figure illustrates the One Health concept via Toxoplasma gondii and arthropod-borne diseases. Blue: Foodborne T. gondii infects humans (e.g., pregnant women) via contaminated meat/produce, potentially causing neurological damage or miscarriage. Green: Cats (definitive hosts) shed oocysts, which contaminate water and soil via rain. Intermediate hosts (e.g., pigs) ingest oocysts; humans risk infection through environmental contact or undercooked meat. Purple: Arthropods (ticks, mosquitoes) cause rashes, fever, itching, and transmit diseases (Lyme, dengue) and pathogens (babesiosis, endemic typhus), embodying the interconnection of human, animal, and environmental health—the core of One Health.

According to the World Health Organization’s 2024 report on Neglected Tropical Diseases (NTDs), parasitic infections remain a devastating global health burden, affecting over a billion people worldwide and causing significant morbidity, mortality, and socioeconomic disruption, particularly in resource-limited regions [1]. Among these, zoonotic parasitic diseases—transmitted between animals and humans—pose a critical and escalating threat. The report emphasizes that factors such as climate change, population migration, and uncertain funding outlooks may lead to the spread of many NTDs beyond their traditional endemic areas in low-income countries and tropical regions [1]. The Coronavirus Disease 2019 (COVID-19) pandemic has fundamentally reshaped global public health awareness, and the “One Health” concept has become a key framework for addressing complex infectious diseases due to its core value in coordinating human–animal-environment health synergy [2]. As demonstrated in a global assessment of schistosomiasis control, environmental strategies that target the intermediate snail host—such as molluscicide application or biological control—reduced disease prevalence by 92 ± 5%. This result significantly outperformed drug-only interventions, which achieved a reduction of only 37 ± 7%. Such evidence highlights the practical impact of the ‘One Health’ framework. Interrupting transmission through ecological management improves control of zoonotic parasitic diseases while reducing dependence on chemical therapies. Consequently, this approach lowers the risk of drug resistance and supports more sustainable disease management [3]. Notably, shifts in vector distribution due to climate change, coupled with health services not yet fully recovering from disruptions caused by the COVID-19 pandemic, are reshaping disease transmission patterns [1]. The report indicates that NTDs cause billions of dollars in losses annually in low- and middle-income countries, while the limitations of traditional treatment options continue to drive this figure upward [1] (Figure 1). Therefore, it is imperative to develop innovative and sustainable solutions to prevent and treat zoonotic parasitic diseases.

Among the various strategies to combat parasitic diseases, transdermal drug delivery systems have received a lot of attention due to their unique advantages [4]. Compared with oral or injectable drug delivery, transdermal drug delivery exhibits several advantages, such as avoiding the first-pass effect of the liver, no gastrointestinal irritation, good compliance, ability to reduce animal stress, convenient use, and high safety [5]. How to make the drug transdermal absorption through the stratum corneum is the focus of the transdermal drug delivery system. Enhancing the permeability of transdermal drugs can be achieved by both physical and chemical methods. Common physical methods include iontophoresis, electroporation, ultrasound introduction, pressure wave, laser cautery, and thermal promotion of permeation, while chemical methods are generally used to enhance transdermal penetration of drugs through chemical permeation promoters [6,7,8]. However, the traditional physical and chemical methods to promote transdermal delivery tend to enhance the rate of transdermal penetration by increasing drug solubility, diffusion coefficient, and reservoir effect, which often leads to the overuse of chemical enhancers and the production of toxic side effects, and thus unmask more drawbacks [9]. Confronted with these difficulties in transdermal drug delivery, nanotechnology may represent a good choice.

Nanotechnology is often defined as the ability to produce and process materials at the nanoscale or to manipulate nano-objects. The nanoscale is often specified as 1 to 100 nm in the field of physics because the electromagnetic and quantum characteristics of nano-objects are more prominent in this scale [10]. Based on the special properties of nano-objects, nanotechnology has been used in various fields of science, and one important application area is nanodrug carriers. Nanodrug carriers include liposomes, transfersomes, ethosomes, and nanoemulsions in which drugs can be encapsulated by dissolution, dispersion, encapsulation, adsorption, and coupling [11]. In the context of transdermal drug delivery, nanodrug carriers offer innovative solutions to overcome the skin’s natural barrier, the stratum corneum, which is a major obstacle to preventing drug penetration. The ex vivo and in vivo behaviors of drugs are determined by the physicochemical properties of the nano carriers, which can be used to enhance drug efficacy, reduce side effects, enhance therapeutic index, and improve drug compliance.

This review focuses on the application of nanotechnology in transdermal treatment systems for parasitic diseases, summarizing its mechanisms of action and the important role it plays in malaria, leishmaniasis, and broad-spectrum antiparasitic drugs. By placing this discussion within the framework of the “One Health” approach, we aim to provide insights that can inform future health policy directions and contribute to the development of innovative and sustainable strategies for combating zoonotic parasitic diseases.

## 2. Advantages of Nano Transdermal Delivery System

### 2.1. Transdermal Mechanism of Nanoformulations

**Figure 2:**
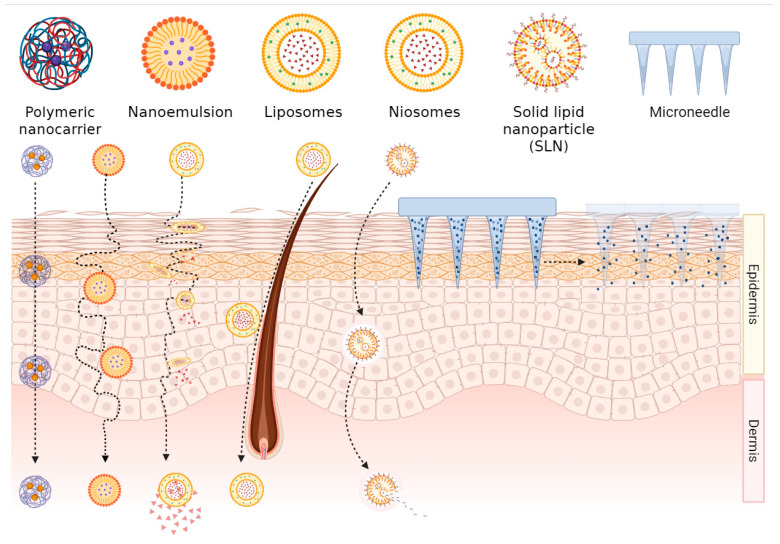
Pathways of transdermal absorption of nanoformulated drugs. This figure illustrates transdermal delivery modalities of nanoformulations. Polymeric nanocarriers, nanoemulsions, liposomes, niosomes, solid lipid nanoparticles (SLNs), and microneedles enable penetration across the skin strata (epidermis and dermis). Leveraging follicular and transcellular/paracellular pathways, these systems exemplify advanced nanocarrier-mediated strategies for percutaneous therapeutic administration.

As the primary physical barrier protecting the human body against external stimuli, the brick-and-mortar structure of the stratum corneum in the outermost epidermal layer severely restricts the transdermal permeation efficiency of conventional drugs through the highly ordered arrangement of intercellular lipids. Nanocarriers break this barrier through a multidimensional synergistic mechanism, significantly enhancing the delivery efficacy of anti-parasitic drugs. The specific mechanisms are as follows and summarized in Figure 2.

The transdermal mechanisms of liposomes involve synergistic effects through multiple pathways: First, the phospholipids in liposomes exhibit high compatibility with stratum corneum lipids, leading to structural disintegration of liposomes and subsequent release of phospholipids. These released phospholipids infiltrate the stratum corneum and reorganize into lamellar structures, thereby widening lipid intercellular channels and enhancing drug permeation [12,13,14]. Second, their hydration effect increases stratum corneum water content, alters the arrangement of intercellular lipid layers in corneocytes, and reduces barrier density. Additionally, intact liposomes can directly penetrate through corneocytes or utilize skin appendage pathways such as hair follicles or sweat glands for delivery [15,16]. Surfactant-driven deformable carriers create transient permeation channels by disrupting stratum corneum lipid organization. Transfersomes are formed by modifying liposomal composition. Surfactants embedded in the vesicle membrane enable extreme deformability, allowing penetration through pores several times smaller than their own size [17,18]. Ethosomes are multilamellar vesicles incorporating high-concentration ethanol into conventional liposomal formulations. The high ethanol concentration enhances the mobility of polar lipid heads in membrane components, thereby increasing lipid fluidity and membrane flexibility. It also reduces the density of intercellular lipid domains, facilitating vesicle deformation during permeation. Consequently, higher ethanol content correlates with greater permeation flux. Drug release and absorption in deeper skin layers result from liposome-skin lipid fusion, which persists throughout the transdermal process [9,19]. For instance, trifluralin-loaded transfersomes demonstrate the potential of deformable nanocarriers for targeted cutaneous leishmaniasis treatment, achieving high-efficiency transdermal delivery without permeation enhancers [20]. Co-loaded nitazoxanide-quercetin nanotransfersomal exhibit a 4-fold enhancement in skin permeation compared to conventional transfersomes [21]. In addition, an ethosomal patch, an innovative antimalarial transdermal nanosystem, has been developed for increasing the cumulative permeation of artesunate by 1.57-fold compared to conventional patches after 8 h of administration [22].

Nanoemulsions significantly enhance transdermal drug permeation through a triple synergistic mechanism. First, surfactant components disrupt the lipid arrangement in the stratum corneum while expanding intercellular spaces through hydration, creating permeation pathways for drug molecules [23]. Second, the nanoemulsion structure enables effective solubilization of drugs, especially lipophilic drugs, thereby increasing the drug concentration gradient across the skin and enhancing permeation [24]. Finally, the nanoscale particle size combined with superior wettability improves skin contact and permeation efficiency [9]. This layer-by-layer mechanism establishes nanoemulsions as ideal transdermal carriers. For example, nanoemulsions effectively deliver chalcone to deep skin layers (dermis), the parasitic site of Leishmania pathogens [25]. Studies have demonstrated enhanced skin permeation using nanoemulsions loaded with C6I/TC1/TC2, which are promising antileishmanial drugs for treating Leishmania infections [26].

Microneedle arrays (MNs), composed of needle-like structures with diameters of several micrometers, penetrate the skin surface to create microchannels, providing an innovative solution for transdermal drug delivery. MNs overcome the limitations of traditional methods through precise targeting capability, offering higher safety, efficacy, and painless administration [27,28,29,30]. For instance, a novel dissolving microneedle patch has been successfully designed to enhance the skin permeability of amphotericin B. The microneedles penetrated rat skin to a depth of 303 ± 8 µm, dissolved rapidly, and formed micropores that caused no significant cellular damage, with natural healing within 30 min [31]. Similarly, a hydrogel-forming microneedle combined with a polyethylene glycol (PEG) reservoir has been developed for cystic Echinococcosis treatment, and in vitro permeation tests showed an albendazole permeation amount of 4584.43 ± 26.61 µg/cm^2^ [32]. Liposomes [14] (including transfersomes [18] and ethosomes [9]), nanoemulsions [23], and microneedle arrays [27] overcome the inherent barrier limitations of the stratum corneum through their distinctive multidimensional synergistic mechanisms—such as the structural compatibility and deformability adaptation of liposomes, the triple synergistic enhancement of permeability in nanoemulsions, and the physical microchannel creation by microneedle arrays. This breakthrough significantly augments the transdermal delivery efficiency of antiparasitic drugs, serving as a critical foundation for subsequent controlled release and targeted delivery systems.

In summary, although liposomes, transfersomes, ethosomes, nanoemulsions, and microneedle arrays all aim to overcome the stratum corneum barrier, their mechanisms and optimal applications differ significantly. Liposomes rely on biocompatibility and fusion with skin lipids, making them suitable for delivering a wide range of drugs, but their penetration efficiency is inherently limited. Transfersomes and ethosomes, as optimized versions, achieve enhancer-free transdermal delivery through superior deformability (imparted by surfactants or high-concentration ethanol, respectively), making them particularly effective for hydrophobic drugs. Nanoemulsions leverage the synergistic effects of surfactant-induced disruption and solubilization, offering greater potential for scalable manufacturing. In contrast, microneedles provide a paradigm-shifting physical strategy that completely bypasses the stratum corneum, enabling the most precise control over drug release and the highest delivery efficiency, albeit with challenges related to production cost and mechanical integrity. Consequently, the selection of an appropriate nanocarrier must be guided by a holistic consideration of drug properties, target disease, production costs, and the intended clinical application.

### 2.2. Controlled Release and Targeted Delivery of Nanoformulations

The success of antiparasitic therapy relies not only on efficient drug penetration through the skin barrier but also on achieving long-acting and low-toxicity therapeutic outcomes through precise controlled-release [33] and targeting strategies [34]. Nanoparticle-based drug delivery systems employ multidimensional collaborative mechanisms to prolong therapeutic duration temporally [35] and enhance lesion targeting efficiency spatially [36], providing comprehensive solutions spanning from localized permeation to dynamic regulation for parasitic infections. This section systematically elaborates the core mechanisms of sustained-release depot construction and targeting synergy, elucidating their pivotal roles in reducing recurrence risk and minimizing host damage.

The sustained-release mechanisms of transdermal nanoformulations achieve high-efficiency antiparasitic therapy through multi-mechanism synergy. Controlled-release carriers such as liposomes [37] and nanoemulsions [34] regulate drug release via matrix diffusion or degradation-controlled processes, forming long-acting drug depots in the skin to reduce dosing frequency. The synergistic interaction between reservoir effects and gradient-driven permeation further optimizes drug delivery: depot-accumulated high-concentration gradients drive drug penetration into subcutaneous tissues, preventing systemic plasma concentration spikes [36]. Building on this, multistage release strategies—employing biphasic rapid-sustained release designs or combination drug loading—enable insecticidal-immunomodulatory synergy, lowering recurrence risks [38,39]. This hierarchical sustained-release system, transitioning from local retention to dynamic permeation and from monotherapy to combinatorial regulation, provides precise long-acting solutions for parasitic infections. Based on this mechanism, a topical ivermectin formulation has been successfully developed, where solid lipid nanoparticles (SLNs) confer superior sustained-release efficacy for treating scabies [40]. Moreover, a novel electrospun wound dressing has been designed and evaluated with excellent antileishmanial activity, achieving sustained drug release for up to two weeks [41,42].

Additionally, hydrogels have emerged as ideal topical drug delivery vehicles. Hydrogels have garnered significant attention due to their unique properties, including low cost, excellent biocompatibility, non-toxicity, stimuli-responsive behavior, and tunable drug release kinetics [43,44]. Nanostructured lipid carrier (NLC)-based hydrogels represent a transformative approach for localized antileishmanial therapy. Inspired by the relevance theory, glucantime-loaded NLC hydrogels have been generated with controlled drug release performance [45].

The passive targeting effect of transdermal nanoformulations primarily optimizes skin penetration efficiency through the physical properties of carriers (e.g., particle size, deformability). For example, trifluralin-loaded transfersomes, with a nanoscale particle size of 140.3 ± 2.3 nm and a high deformation index (DI = 43.5 ± 1.0), can permeate through microscopic pores in the stratum corneum [20]. Additionally, the negatively charged surface of TFS may enhance interactions with macrophage phagosomal membranes, promoting intracellular drug delivery [20]. Experimental validation revealed that rhodamine-labeled TFS exhibited a 4.13-fold increase in endocytosis efficiency compared to free solutions, directly attributed to the adaptive membrane penetration of their elastic vesicular structures. This property demonstrates significant advantages in antileishmanial therapy: trifluralin-loaded transfersomes achieved 90.87% inhibition of intracellular amastigotes in macrophages at 50 µg/mL, showing a 1.68-fold efficacy enhancement over conventional TFL solutions that exhibited 54% inhibition [20]. In a similar strategy, rifampicin-loaded nanotransfersomes also rely on nanoscale size and elasticity for passive targeting, further validating the universality of this mechanism in transdermal antiparasitic therapy [46]. Furthermore, elastic liposomes are demonstrated to enhance immune responses via transdermal delivery of the Plasmodium falciparum surface antigen (PfMSP-1_19_), effectively activating Langerhans cells and promoting their migration to draining lymph nodes [47,48].

The deep integration of these mechanisms and advantages marks the transition of nanocarrier-based transdermal delivery systems into a new era of precision and long-acting performance in antiparasitic therapy. Currently, this technology has displayed significant clinical value in zoonotic parasitic diseases such as leishmaniasis and malaria. Its specific applications and future challenges will be systematically discussed below.

## 3. Application of Nanoformulations in the Treatment of Parasitic Diseases

Nanotechnology-based systems, leveraging their unique technological advantages, provide highly promising solutions for antiparasitic drug delivery. The application of nanocarriers significantly enhances drug targeting specificity, effectively protects drugs or immunotherapeutic agents from extracellular degradation, and optimizes biopharmaceutical and pharmacological properties, thereby improving therapeutic efficacy while reducing side effects [49]. Currently, nanocarrier-based therapy has been extensively used in the control of parasitic diseases, among which Leishmaniasis and Malaria are the most noteworthy aspects. Herein, we take Leishmaniasis and Malaria as examples to discuss the application of nanoformulations for parasitic disease control.

### 3.1. Leishmaniasis

Leishmaniasis, caused by protozoan parasites of the genus Leishmania (family Trypanosomatidae), is transmitted through the bite of infected sand flies, resulting in a spectrum of clinical manifestations including visceral (VL), cutaneous (CL), mucosal (ML), and mucocutaneous leishmaniasis (MCL) [50,51]. The severity of illness is determined by the virulence of specific Leishmania species and the host immune response, ranging from self-resolving cutaneous lesions to life-threatening visceral organ failure [51,52,53]. Macrophages, serving as the primary host cells for Leishmania parasites, play a pivotal role in pathogenesis. Despite substantial research efforts, challenges such as drug resistance and limited therapeutic accessibility persist, necessitating accelerated development of novel therapies to address this ongoing global health burden [54].

Current pharmacotherapy for Leishmaniasis faces multifaceted limitations, including drug toxicity, pathogen resistance, and restricted access to treatment resources, particularly in low-income regions. First-line pentavalent antimonials have been progressively phased out due to widespread resistance [55,56]. Alternative agents such as miltefosine, paromomycin, and pentamidine are further constrained by emerging resistance, prohibitive costs, and severe adverse effects. In light of these challenges, nanotechnology-driven formulations have emerged as a promising strategy to improve drug efficacy and reduce toxicity for both visceral [57] and cutaneous leishmaniasis [58]. Particularly noteworthy is that nanoformulations can enhance the therapeutic efficacy of existing pharmacophores, such as quinoline compounds, by improving their delivery and targeting capabilities [59]. Herein, we systematically examine recent advances in therapeutic strategies against Leishmaniasis, focusing on transdermal penetration enhancement, safety optimization, wound healing promotion, and immune modulation, while addressing translational challenges and future research trajectories.

Transdermal delivery technologies have undergone multistage iterative optimization. Early research focused on nanoemulsions: a chalcone nanoemulsion has been developed using a mixed system of anionic and nonionic surfactants, which enhanced dermal permeation of trans-chalcone and 3′-(trifluoromethyl)-chalcone by 1.26-fold and 2.41-fold, respectively [25,60,61]. The fluorinated derivative achieved 88.31 μg/g skin retention (vs. 45.86 μg/g for the free form) due to fluorine-enhanced hydrophobicity [25]. Garcia et al. further validated this potential: an ethanol-oleic acid/Tween^®^-80 nanoemulsion with particle size modulation of 147–273 nm enabled sustained release of anti-leishmanial compounds (first-order kinetics and Weibull model), demonstrating 2-fold increased parasiticidal activity and reduced macrophage cytotoxicity [26]. Recent advances highlight deformable nanocarriers for enhancer-free transdermal delivery. Second-generation systems achieve precision delivery through structural engineering. A prime example is trifluralin-loaded transfersomes, which have a particle size of 140 nm and an ultralow polydispersity index (PDI, 0.006). This achieved 86% encapsulation efficiency and 24 h sustained release without permeation enhancers [20]. This reduced Leishmania IC_50_ values by 2.86 to 3.07-fold, suppressed amastigotes by 90.87% (vs. 54% for free drug), and demonstrated efficient macrophage internalization and biocompatibility [20]. Furthermore, it is reported that nitazoxanide-quercetin nanotransfersomal gel (NTZ-QUR-NTG) enhanced skin permeation 4-fold, increased macrophage uptake 10-fold, elevated concentration of cytotoxicity 50% (CC_50_) from 49.77 μg/mL to 71.95 μg/mL, and significantly reduced lesion size in mice with minimized systemic toxicity [21]. Interestingly, microneedle technology physically bypasses the stratum corneum barrier. Based on this mechanism, a PVP-carboxymethylcellulose microneedle patch has been successfully developed with 303 ± 8 μm penetration depth, releasing amphotericin B within 30 min through self-sealing micropores, achieving 86% anti-leishmanial activity [31]. Additionally, engineered electrospun core–shell nanofibers have been demonstrated to sustainably release 84% glucantime over 9 h, maintaining therapeutic drug concentrations for prolonged efficacy [62].

**Figure 3:**
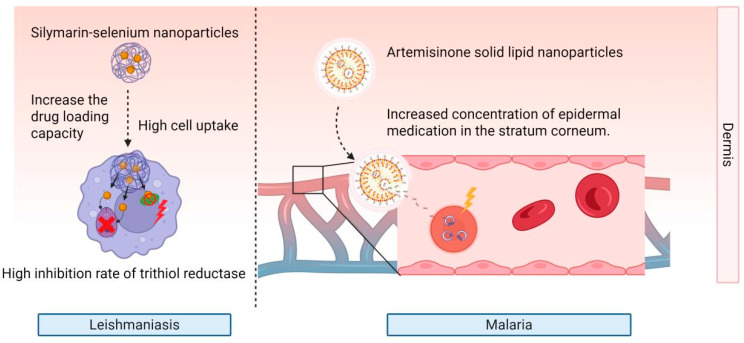
Examples for transdermal nanoformulations against leishmaniasis and malaria. This figure illustrates nanotherapeutic mechanisms against Leishmaniasis (left) and Malaria (right). Silymarin-selenium nanoparticles enhance drug loading, promote cellular uptake, and inhibit trithiol reductase for Leishmaniasis. Artemisininone solid lipid nanoparticles boost epidermal drug concentration in the stratum corneum to treat Malaria, showcasing nano-based strategies for parasitic diseases.

To enhance the safety profile and immunomodulatory efficacy of nanoformulations, breakthroughs have been achieved across multiple dimensions. In nanomaterial innovation, silymarin-selenium nanoparticles exhibit a skin deposition rate of 82.21%, which reduced local toxicity by inhibiting trypanothione reductase activity and improving cytocompatibility [63] (Figure 3). The Rabia team has engineered rifampicin-loaded nanotransfersomes combined with a chitosan-based gel, achieving 3-fold enhanced skin permeability and reduced IC_50_ values through passive macrophage targeting, thereby minimizing systemic exposure while boosting therapeutic efficacy via apoptosis induction [46]. In novel delivery system design, a glucantime-loaded nanostructured lipid carrier (NLC) hydrogel has been formulated that enabled sustained drug release, enhanced skin retention in murine models, and, compared to injectable formulations, this system significantly reduced lesion size, parasite burden, and systemic toxicity, offering a non-invasive therapeutic strategy [45]. For immune modulation, Lanza et al. pioneered a LiHyp1 recombinant antigen-based vaccine delivered via dissolving microneedles (DMNs) and cationic liposomes (CL). This approach elicited high-titer antibody responses in mice, suppressed L. donovani infection, and established a novel paradigm for anti-leishmanial immunotherapy [64].

Functionalized nanomaterials synergize intelligent drug release with multimodal mechanisms to promote chronic ulcer repair. On the basis of this mechanism, engineered chitosan-polyethylene oxide nanofibers loaded with berberine have been successfully designed, achieving gradient release over 14 days (80% released within the first 3 days). This system demonstrated potent larvae growth inhibition (IC_50_ = 0.24 µg/mL) and excellent biocompatibility [41]. Furthermore, its efficacy has been validated in accelerating Leishmania ulcer healing, noting stable berberine release kinetics coupled with dual antibacterial activity against Staphylococcus aureus and Escherichia coli and sensor compatibility for real-time wound microenvironment monitoring [42]. Similarly, a PVP-PVA-clay hydrogel has been shown to achieve 15 h sustained release of N-methyl glucamine under 1.5% clay conditions, reducing lesions by 99% in L. amazonensis-infected models [65]. A cobalt-60 radiation-crosslinked PVP-PEG-agar hydrogel loaded with amphotericin B exhibited 12 h continuous drug release with thermal/radiation stability, achieving 100% parasitic growth inhibition within 48 h while integrating sterilization and long-term anti-infection functions [66].

Nanoparticle-based transdermal delivery technologies, through strategies such as deformable nanocarriers, microneedle patches, and smart hydrogels, have significantly enhanced transdermal efficiency and targeting specificity in the treatment of Leishmaniasis, while reducing systemic toxicity via sustained-release designs. However, their clinical translation remains constrained by manufacturing complexity, insufficient long-term biosafety validation, and accessibility challenges in resource-limited regions. Future efforts should prioritize the development of intelligent responsive systems (e.g., enzyme/pH dual-sensitive hydrogels), artificial intelligence (AI)-driven carrier optimization, and multimechanistic combination therapies. Concurrently, leveraging global health governance frameworks such as the “One Health” initiative will be critical to advancing technological equity and accelerating the elimination of cutaneous leishmaniasis.

### 3.2. Malaria

Malaria is a major infectious disease that causes severe morbidity, mortality, and economic burden globally [67]. Although significant efforts have been made to eradicate Malaria, the number of malaria cases worldwide still reached 249 million in 2022, resulting in 608,000 deaths [68]. The burden of Malaria remains particularly severe in countries such as Nigeria, Uganda, the Democratic Republic of the Congo, and Mozambique [68]. Malaria is caused by a single-celled parasite known as Plasmodium, which is transmitted to humans through the bites of female Anopheles mosquitoes, continuously threatening the lives of millions of people worldwide. There are five known species of Plasmodium that can cause human malaria, including Plasmodium falciparum, Plasmodium malariae, Plasmodium vivax, Plasmodium ovale, and Plasmodium knowlesi, with Plasmodium falciparum having the highest mortality rate [69].

The treatment of malaria mainly depends on chemical drugs such as chloroquine, lumefantrine, primaquine, artemisinin, and its derivatives. While these drugs have achieved certain effectiveness in controlling malaria, their effectiveness is increasingly influenced by various factors, including the emergence of multidrug resistance, nonspecific targeting of intracellular parasites, low solubility and bioavailability of the drugs, and significant toxicity of the medications [70,71]. Increasing resistance manifests itself in the ability of the parasite to survive and multiply at a certain concentration of the drug that would normally be killed, so it is important to seek drug combinations and new strategies. The WHO recommends the use of artemisinin-based combination therapies (ACTs) for the treatment of malaria, including artesunate-amodiaquine, artemether-lumefantrine, artesunate-sulfadoxine-pyrimethamine, dihydroartemisinin-piperaquine, and artesunate-mefloquine [72]. However, they also face issues such as high costs, differences in drug metabolism characteristics, potential toxicity, and insufficient availability [72]. Furthermore, problems such as high transmission rates of Plasmodium parasites, resistance of mosquito vectors to insecticides, logistical challenges in implementing control measures, and population mobility pose greater difficulties for malaria control efforts. To overcome these difficulties, in recent years, innovations in nanoparticle-based drug delivery technology have provided breakthrough solutions [68]. Next, we systematically review relevant advances, focusing on three key dimensions: enhancement of targeting specificity, reversal of drug resistance, and mitigation of toxicity, aiming to provide scientific evidence for optimizing antimalarial strategies.

Improving drug targeting specificity represents a core breakthrough direction in nanotechnology. Ethosomes containing artesunate and febrifugine into a patch matrix to form a composite transdermal patch have been prepared, achieving 8 h cumulative permeation 1.57 times higher than conventional patches, with 100% parasite clearance and zero recurrence, demonstrating the high efficiency of transdermal delivery [22]. The transdermal nanogel platform exemplifies this progress, as its tunable size, adhesive properties, and sustained-release capabilities can effectively enhance drug retention in the skin layers and improve local targeting, offering a novel strategy for antimalarial therapy [73]. The Zech team further optimized an artemisinin-based microemulsion spray [74]. In a cerebral malaria model, this formulation achieved a blood concentration exceeding 500 ng/mL, doubled the efficacy compared to artesunate microemulsions, and enabled complete pathogen eradication [74]. Similarly, artemisone-loaded solid lipid nanoparticles, through precise regulation of particle size and crystallinity, elevated drug concentration at the stratum corneum-epidermis junction to 62.632 μg/mL (versus 12.792 μg/mL for artemisone-loaded niosomes), demonstrating the significant efficacy of nanoparticles in optimizing dermal drug delivery concentration [75] (Figure 3). In the field of immunomodulation, an elastic liposomal vaccine delivering Plasmodium falciparum antigen PfMSP-119 has been developed, which markedly enhanced humoral and cell-mediated immune responses [48], maintained peak IgG-specific antibody titers for 70 days post-immunization, exhibited more sustained immune responses compared to conventional liposomes and intramuscular injection groups, and significantly increased IFN-γ secretion levels (approximately 9-fold higher than controls) [47]. This immuno-based system provides a novel strategy for developing needle-free malaria vaccines [47,48].

To combat drug resistance, scientists have significantly enhanced therapeutic efficacy through co-delivery systems and novel mechanism designs. Aderibigbe’s team has developed an Arabic hydrogel dual-drug loading system co-encapsulating 4-aminoquinoline and curcumin that, by leveraging differential release kinetics (short-term anomalous transport and long-term quasi-Fickian diffusion), achieved synergistic effects, providing a new strategy for antiparasitic combination therapies [38]. Further studies revealed that carbomer-polyacrylamide-soy protein composite hydrogels, through temporal regulation of chloroquine and curcumin diphosphate release via a super case II transport mechanism, enabled phased drug release to overcome current antimalarial drug resistance challenges [39]. To address the limitations of resistance to conventional drugs, a hydroxypropyl methylcellulose-polyvinylpyrrolidone hydrogel nanoparticle loaded with curcumin has been successfully designed and prepared. This nanoparticle significantly enhanced curcumin’s antimalarial activity (exhibiting doubled potency compared to free drug at 25 mg/kg), prolonged drug circulation time, and improved bioavailability for enhanced efficacy [76]. Moreover, Mavondo’s team has developed a pectin hydrogel-based percutaneous patch that not only suppresses parasitemia but also ameliorates inflammatory responses and anemia symptoms in malaria-infected Sprague-Dawley rats, offering a multifunctional solution for drug-resistant malaria treatment [77].

In addressing systemic toxicity reduction, nanotechnology strategies focusing on localized controlled release and biocompatibility optimization have demonstrated remarkable efficacy. Nnamani et al. developed an artemether-loaded nanostructured lipid carrier hydrogel, which forms a localized drug reservoir through transdermal penetration, achieved a 26% cumulative permeation rate over 48 h, significantly enhanced drug stability, release controllability, and demonstrated no observable systemic exposure [78]. Notably, the transdermal route itself possesses an inherent capacity to circumvent common gastrointestinal side effects and first-pass metabolism associated with oral antimalarials, enabling a source reduction in systemic toxicity risks, a feature particularly pronounced in novel transdermal formulations like nanogel and film [72]. Conventional oral administration of primaquine (PMQ) may induce adverse effects and undergo significant first-pass metabolism in the liver, thereby compromising therapeutic efficacy. Ananda’s team pioneered the exploration of PMQ transdermal patch technology combined with solid microneedles (Dermaroller^®^) [79]. Their comprehensive evaluation included multiple parameters such as thickness, weight uniformity, and pH values. In vitro and ex vivo tests on PMQ release and permeation through rat skin confirmed the system’s safety profile, with all formulations demonstrating optimal performance, establishing a safe paradigm for long-term medication [79].

Looking forward, although nanoparticle-based drug delivery technologies have demonstrated advantages in targeting specificity and safety profiles, their large-scale application still requires overcoming cost constraints and manufacturing barriers to provide sustainable technological support for global malaria eradication. Beyond the prominent examples of leishmaniasis and malaria, the versatility of nanotransdermal delivery systems holds significant promise for addressing a broader spectrum of zoonotic parasitic infections. This potential is further amplified by the convergence of nanotechnologies across different administration routes, a pivotal development exemplified by the success of nanoformulations for diseases like Chagas disease via oral or parenteral routes [80]. Together with the advances in transdermal delivery detailed here, this collectively represents an overarching ‘paradigm shift’ towards nano-enabled therapies. In the specific context of transdermal delivery, helminthic infections such as cystic echinococcosis [81] and lymphatic filariasis [82] face challenges similar to those discussed, including poor drug bioavailability, systemic toxicity, and the need for prolonged therapy. As highlighted in section four, nanotechnological approaches, particularly microneedle-integrated systems, demonstrate remarkable efficacy in enhancing transdermal delivery, achieving targeted deposition (e.g., lymphatic targeting), and enabling sustained release, thereby improving therapeutic outcomes while mitigating adverse effects [29,83]. Other neglected tropical diseases with zoonotic components, such as onchocerciasis and strongyloidiasis, could similarly benefit from the advancements in nanocarrier design (liposomes, nanoemulsions, SLNs) tailored for transdermal delivery of ivermectin, albendazole, or other relevant anthelmintics [84,85]. The core principles of overcoming the stratum corneum barrier, enhancing local drug concentration, enabling controlled release, and reducing systemic exposure are universally applicable. The success seen with leishmaniasis, malaria, and broad-spectrum drugs provides a strong foundation for expanding the application of this technology across the neglected zoonotic parasitic disease landscape.

## 4. Nanoformulations in Broad-Spectrum Antiparasitic Drugs

Beyond targeted therapies against specific pathogens (e.g., Leishmania, Plasmodium), nanoparticle-based transdermal delivery technologies have provided revolutionary solutions for enhancing the application of conventional broad-spectrum antiparasitic drugs. Taking avermectin and albendazole as examples, both exhibit high efficacy against a wide range of endo- and ectoparasites; however, their conventional administration routes, such as oral administration, are limited by low bioavailability, systemic toxicity, and poor patient compliance [32,40]. Nanoparticle-based reformulation significantly enhances transdermal permeation efficiency, targeting specificity, and therapeutic window of avermectin- and Benzimidazole-class drugs. We next review and discuss the updates related to nanoformulation-driven anthelmintic drugs (Table 1).

### 4.1. Avermectin-Class Drugs

Avermectin-class drugs such as avermectin, ivermectin and eprinomectin play an indispensable role in veterinary and human parasitic disease control due to their exceptional broad-spectrum antiparasitic activity [91]. For instance, ivermectin represents a landmark therapeutic for treating onchocerciasis [84], lymphatic filariasis [92], scabies [93], and strongyloidiasis [85], and is included in the World Health Organization Essential Medicines List [88]. However, they have long been constrained in their antiparasitic efficacy due to low solubility, insufficient transdermal permeation efficiency, and systemic toxicity risks [88]. Multifaceted innovations in nanoparticle-based delivery technologies—through formulation design optimization and enhancement of controlled-release strategies—are progressively advancing their translation from laboratory research to clinical practice.

**Figure 4:**
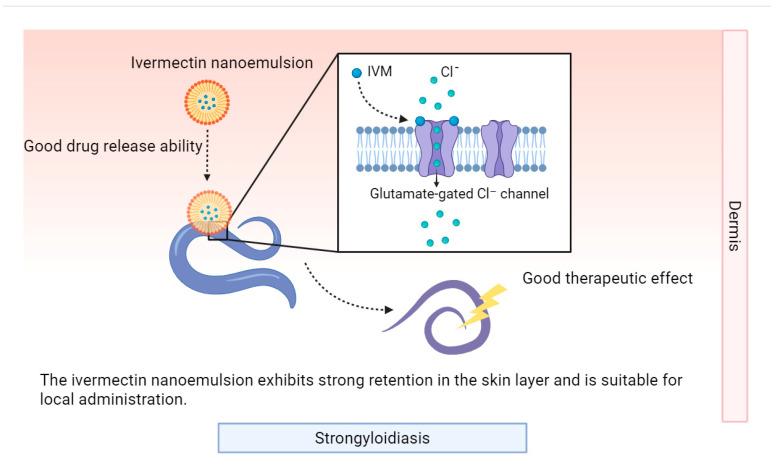
Mechanism of ivermectin nanoemulsion in treating strongyloidiasis.

Early studies focused on solubility optimization and enhancement of the transdermal permeation efficiency of avermectin-class drugs. For instance, Das et al. pioneered a breakthrough with ivermectin-loaded microemulsion-based gel formulations using nanoscale technology, where in vitro membrane permeability assays demonstrated superior permeation advantages, enabling low-dose drugs to achieve equivalent or better efficacy compared to conventional formulations [86]. Subsequently, an advanced ivermectin nanocrystal technology through nanocrystallization combined with lyophilization, achieved a 730-fold increase in equilibrium solubility, a 24-fold acceleration in dissolution rate, and threefold higher drug deposition in the deep dermis, significantly reducing side effect risks [87]. Building upon these advancements, formulation diversity exploration has enabled tailored solutions for different infection scenarios. A recent study systematically compared three ivermectin-loaded formulations: nanoemulsion (NE), nanoemulsion gel (NEG), and colloidal system (CS) [88]. NE exhibited the highest drug concentration at the stratum corneum and epidermis/dermis junction, making it ideal for localized therapy. NEG demonstrated superior adhesiveness, solubilization capacity, and occlusiveness, achieving the fastest delivery rate. CS showed the highest drug diffusion percentage, enabling efficient delivery via the follicular route at a 0.35% low dose (versus 2% in other systems) [88]. This study not only highlights formulation design diversity but also provides a theoretical foundation for precision therapy by using conventional antiparasitic drugs (Figure 4).

To balance efficacy and safety of antiparasitic drugs, controlled-release technology has become a key research focus. Ivermectin-loaded solid lipid nanoparticles exhibiting excellent sustained-release efficacy, with their targeting capability significantly reducing systemic toxicity and side effects [40]. With the acceleration of technology development, Mao et al. designed hybrid micelles that demonstrated 1.92-fold higher transdermal efficiency compared to the commercial pour-on formulation (Eprinex^®^), and in vivo trials support its favorable safety profiles [90]. In addition, the release profiles of mesoporous silica nanoparticles (IVM-MCM) and polymeric nanocapsules (IVM-NC) have been further studied. IVM-MCM facilitates rapid drug release suitable for acute infection management, whereas IVM-NC provides sustained release ideal for chronic prevention requirements [89]. This rational optimization of ivermectin nanoformulations provides a novel strategy to accelerate its clinical translation [89].

From solubility breakthroughs in microemulsion-based gels [86] to transdermal optimization of hybrid micelles [90], and from explorations in formulation diversity [88] to the modulation of release kinetics [40]. Avermectin-class nanoformulations are progressively overcoming some of the key limitations inherent in conventional formulations. However, their full clinical translation still requires overcoming barriers in manufacturing scalability, safety profiles, and therapeutic validation. Going forward, cross-disciplinary collaboration and technological innovation will be pivotal to translating the theoretical advantages of nanoformulations into practical clinical benefits for conventional anthelmintic drugs.

This figure illustrates the mechanism of ivermectin nanoemulsion (IVM-NE) in treating strongyloidiasis. IVM-NE, with favorable drug-release ability, releases ivermectin (IVM). IVM acts on glutamate-gated Cl^−^ channels of Strongyloides organisms. It shows strong dermal retention, good safety, and is suitable for topical administration to combat strongyloidiasis.

### 4.2. Benzimidazole-Class Drugs

Benzimidazole-class drugs constitute the cornerstone of antihelminthic therapy [94], exhibiting highly effective broad-spectrum activity against diverse intestinal nematodes, cestodes, and certain trematodes [95]. However, oral formulations of conventional benzimidazoles are severely limited in antiparasitic efficacy and safety due to poor aqueous solubility (only 48.11 μg/mL in PBS), significant first-pass metabolism (bioavailability of less than 5%), and hepatotoxicity induced by systemic exposure [32]. Recent innovative strategies based on nanotechnology and transdermal delivery systems have successfully overcome these limitations, with key advancements manifested in the following aspects.

First, drug morphology reconstruction using carriers like solid lipid nanoparticles, the solubility kinetics of albendazole have been significantly improved. Permana et al. formulated albendazole into nanocrystals, achieving 89.92% cumulative drug release over 24 h in vitro with a 3.2-fold accelerated release rate [81]. Similar to that, a polyethylene glycol reservoir stabilizes albendazole in an amorphous state, achieving a solubility of 283.62 μg/mL, representing a 5-fold increase [32]. Therefore, nanonization not only overcomes benzimidazole’s intrinsic hydrophobicity but also enables rapid therapeutic onset through accelerated dissolution.

**Figure 5:**
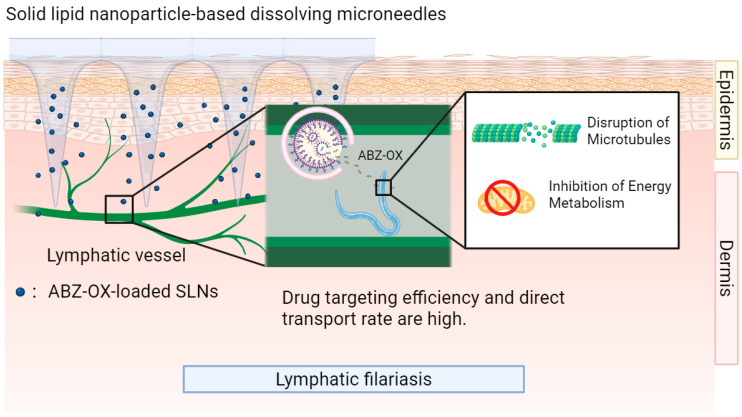
Mechanism of solid and lipid nanoparticle-dissolving microneedle system of albendazole. This figure illustrates the mechanism of solid lipid nanoparticle (SLN)—based dissolving microneedles for lymphatic *filariasis* treatment. ABZ-OX-loaded SLNs penetrate the skin via microneedles and target lymphatic vessels. Advantages include high drug-targeting efficiency and direct transport rate, disrupting microtubules and inhibiting the energy metabolism of filarial parasites for effective therapy.

Second, the application of dissolving microneedles (DMNs) and hydrogel-forming microneedles (HFM) has overcome skin barrier limitations, enabling precision drug delivery. Based on this nanoformulation, an albendazole-loaded dissolving microneedles-nanocrystals system achieved a penetration depth of 505.29 μm in ex vivo porcine skin, with a peak dermal drug concentration of 1891.53 μg/cm^3^ [81]. For albendazole sulfoxide delivered via a solid lipid nanoparticle—dissolving microneedle system, both drug targeting efficiency and direct transport percentage were significantly enhanced, reaching 11.99-fold and 91.66%, respectively [83] (Figure 5). Additionally, the HFM-PEG integrated system delivering albendazole demonstrated a 24 h transdermal permeation of 4584.43 μg/cm^2^—tens of times higher than oral formulations [32]. Taken together, microneedle technology facilitates localized high-concentration exposure and lymphatic-targeted uptake, directly acting on parasite habitats (e.g., subcutaneous or lymphatic systems) while bypassing systemic metabolic losses.

Third, the sustained-release design of solid lipid nanoparticles and nanocrystals significantly prolongs drug action duration. Permana et al. demonstrated that the dissolving microneedles-nanocrystals system extended the drug half-life to 136 h (versus 3.54 h for oral nanocrystals) [81]. The SLN-based system achieved 65.35% sustained release of albendazole sulfoxide (ABZ-OX) over 48 h, while reducing the generation of metabolite ABZ-ON by 65%. Furthermore, drug distribution levels in the liver and kidneys were only 27.5% and 44.5%, respectively, compared to the oral administration group [83]. Mahfufah’s study further revealed that the nanosystem exhibited a hemolysis rate of less than 5%, maintained skin integrity, and a surface pH of 5.43–5.51, compatible with the physiological pH range, demonstrating significantly enhanced safety over conventional formulations [32].

Collectively, nanotransdermal formulations for benzimidazole class drugs through a synergistic mechanism integrating ‘dissolution enhancement, delivery potentiation, and toxicity control’ have achieved a breakthrough in both relative bioavailability and lymphatic targeting efficiency, providing highly effective, long-acting, and low-toxicity innovative solutions for cystic echinococcosis [32,81] and lymphatic filariasis [83]. Future efforts should prioritize advancing clinical translation trials to evaluate the feasibility of scaled-up production and long-term safety profile.

## 5. Summary and Perspectives

Nanoparticle-based transdermal drug delivery systems, leveraging their unique delivery mechanisms and precisely controlled-release capabilities, have pioneered revolutionary pathways for antiparasitic drug development. Nanoformulations, including liposomes, nanoemulsions, and microneedles, nanocarriers overcome the physical barrier of the stratum corneum, significantly enhancing local drug permeation efficiency and targeting specificity, while decreasing first-pass metabolism and systemic toxicity associated with conventional administration routes. However, translating these promising nanoparticle-based formulations from laboratory research to clinical application still requires overcoming interrelated challenges in technology, production, and clinical translation.

Current challenges primarily revolve around the conflict between skin barrier penetration efficiency and targeting precision. The complex structure of the skin and the dynamic physiological microenvironment may compromise the stability of nanocarriers and their efficiency in achieving precise drug release. Moreover, the multi-stage life cycle and tissue tropism characteristics of parasites necessitate highly intelligent targeting strategies. Current environmentally responsive carriers, such as pH-sensitive systems, still face challenges in ensuring that their release kinetics fully align with the pathological microenvironmental fluctuations at the infection site.

Beyond technical performance, scalable manufacturing and rigorous safety evaluation remain significant bottlenecks. Issues such as batch-to-batch variability in encapsulation efficiency, insufficient long-term storage stability, and complex quality control standards pose major obstacles to industrial production. In resource-limited regions, high production costs and low-temperature storage requirements (e.g., lyophilization processes) further limit technological accessibility. Meanwhile, the long-term biosafety of nanomaterials remains incompletely characterized. Potential risks such as dermal irritation, immunogenicity, and systemic accumulation effects require validation through more comprehensive evaluation systems, including 3D skin models and computational toxicology.

Most critically, its clinical translation and commercialization pathway still require substantial refinement, particularly in providing more detailed evidence regarding clinical trials, regulatory approval, and economic viability. The current evidence base is primarily built on in vitro and preclinical animal studies, while large-scale clinical trial data necessary for advancing to clinical application remain scarce. The regulatory approval pathway for nanomedicines is complex and lacks a fully standardized framework, requiring comprehensive characterization and strict review by agencies such as the FDA and EMA regarding the in vivo behavior, stability, and potential toxicity of nanoformulations. Finally, economic viability is crucial in determining its accessibility. The high costs associated with complex materials, sophisticated manufacturing processes, and potential cold-chain storage requirements hinder the application of this technology in low-income regions where it is most needed.

The underlying root cause of the aforementioned economic feasibility challenges lies in a core contradiction within the public health domain. While malaria and leishmaniasis receive prioritized support from international organizations, nanopharmaceutical research and development for most neglected tropical diseases (NTDs) (e.g., onchocerciasis, schistosomiasis) remains reliant on non-profit funding with weak commercial drivers. Strategically leveraging policy instruments (e.g., the Medicines Patent Pool, MPP) and regional collaborations to reduce costs, coupled with advancing generic drug manufacturing at scale, has become pivotal to achieving equitable technological accessibility.

Therefore, to systematically address the multi-faceted challenges described above, it requires not only continuous innovation in fundamental research but also hinges critically on the rational selection of nanoplatforms tailored to specific application contexts. For instance, functionalized liposomes and transfersomes, with their superior intracellular delivery capabilities, tend to be the preferred candidates for managing intracellular infections like leishmaniasis. In contrast, for broad-spectrum anthelmintics that require high-dose and long-term administration, scalable nanoemulsions or sustained-release nanocrystals may offer more practical advantages due to their manufacturing feasibility. Meanwhile, microneedle technology holds unique potential for one-off vaccination or precise lymphatic targeting applications, albeit at a higher unit cost. This strategic selection process itself is a critical step toward viable applications.

Looking forward, breakthroughs in nanotechnology-based transdermal drug delivery systems will require multidimensional innovation strategies. At the scientific frontier, the development of bioinspired nanocarriers (e.g., parasite membrane-camouflaged systems) and intelligent responsive materials (e.g., thermo/enzyme-sensitive hydrogels) holds promise for achieving spatiotemporal dynamic drug release. The integration of microneedle arrays with nanotechnology could mechanically breach the skin barrier while enabling precise modulation of drug release kinetics. Technologically, artificial intelligence (AI)-driven carrier design (e.g., machine learning-optimized lipid compositions) and modular manufacturing processes (e.g., microfluidic continuous-flow technology) are expected to significantly shorten research and development cycles and reduce costs. Furthermore, advancing the global health governance framework—particularly through the “One Health” initiative—could accelerate technology translation via public–private partnerships, while developing region-adapted formulations (e.g., prefilled microneedle patches) for low-resource settings.

Transdermal delivery of nanoformulation represents not merely a technological innovation but a critical practice in advancing global health equity. Only by overcoming the systemic barriers among penetration efficiency, targeting precision, manufacturing feasibility, and health policy alignment can we translate this potential into life-saving therapeutics, ultimately advancing the vision of the elimination of neglected tropical diseases.

## Figures and Tables

**Table 1:** Nanoformulations of antiparasitic drugs for zoonotic diseases.

Indication	Nanoformulation	Compounds	Advantages	Study Type and Model	Formulation Characteristics	Year	References
Leishmaniasis	Transfersomes	Trifluralin	High transdermal efficiency, high encapsulation efficiency, sustained release, reduced IC_50_ for Leishmania pathogens, high inhibition rate of amastigotes	In vivo (Albino Wistar rats)	PS: 140.3 ± 2.3 nm; PDI: 0.006 ± 0.002; %EE: 86 ± 0.5% and 43.5 ± 1.0%	2022	[20]
	Transfersomes	Nitazoxanide-quercetin	Enhanced skin permeation, increased macrophage uptake, higher CC50, smaller lesion size, low systemic toxicity	In vitro (PMs) In vivo (Male Albino Wistar rats, BALB/c mice)	PS: 210 nm; PDI: 0.16; ZP: −15.1 mV; EE of NTZ and QUR was 88% and 85%.	2023	[21]
	Nanoemulsions	3′-(Trifluoromethyl)-chalcone	High dermal permeation amount, high skin retention	In vitro (TH-1) in vivo (Pigs)	PS: 179.0 ± 1.0 nm; PDI: <0.3; %EE: ~100%; |ZP|: > 30 mV	2021	[25]
	Nanoemulsions	C6I/TC1/TC2	Enhanced skin permeation, Sustained release, high parasiticidal activity, low macrophage cytotoxicity	In vitro (U-937-CRL-1593.2)	/	2023	[26]
	Electrospun core–shell nanofibers	Glucantime	Stable drug release, maintenance of effective drug concentration for a long time, sustained therapeutic effect	In vitro (NIH3T3, Franz diffusion cell)	/	2020	[62]
	Nanoparticles	Silymarin-selenium	High drug loading capacity, high skin deposition rate, significant reduction in local treatment toxicity	In vitro (NHDFa) In vivo (Male Wistar rats)	%LE: 58.22 ± 0.56%; HD: 245.77 ± 11.12 nm; PDI: 0.19 ± 0.01; ZP: −30.63 ± 0.40 mV	2024	[63]
	Transfersomes	Rifampicin	High skin permeability, targeted reduction of IC_50_ value	In vitro (Macrophages) and in vivo (Albino Wistar rats, female BALB/c mice)	PS: 190 nm, %EE: 83%; 3-fold permeation vs. free RIF.	2020	[46]
	Nanostructured lipid carrier	Glucantime	Controlled drug release, enhanced skin retention, reduced systemic toxicity	In vitro (L. major) and in vivo (female BALB/c mice)	PS: 93.87 ± 0.1 nm; PDI: 0.295 ± 0.015; ZP: −30.31 ± 0.25 mV; %LE: 74 ± 0.37%	2023	[45]
	Nanofibers	Berberine	Gradient release, excellent biocompatibility, stable release rate	In vitro (J774A.1, L. major) and in vivo (female BALB/c mice)	PS: 10.51 ± 0.24 nm; PDI: 0.19 ± 0.03; ZP: −0.41 ± 0.17 mV	2023, 2024	[32,33]
	Microneedle	Aphotericin B	Improved skin permeability, minimal cellular damage	In vitro (HT-29,) In vivo (Sprague-Dawley male rats)	Drug Loading: 182 ± 4 μg per (22 × 22) MN array	2021	[31]
*Malaria*	Ethosomes	Artesunate and Febrifugine	High cumulative permeation, high efficiency	In vivo (Specific-pathogen-free male Kunming mice)	PS: 26.48 ± 0.12 nm; ZP: −28.0 ± 1.6 mV; PDI: 0.195 ± 0.005; %EE: 71.81 ± 2.57%	2015	[22]
	Solid lipid nanoparticles	Artemisone	High skin delivery concentration	In vitro (Caucasian female skin obtained by abdominoplasty)	PS: 295 ± 18 nm; ZP: −12 ± 3 mV; %EE: 79 ± 5.00%	2016	[75]
	Elastic liposomes	PfMSP-119	Efficient targeting, long-lasting immune response	/	/	2016	[49]
	Nanoparticles	Curcumin	High antimalarial activity, prolonged drug circulation time, high bioavailability, enhanced therapeutic efficacy	Albino nude rat, Balb/c mice	/	2015, 2016, 2010	[47,48,76]
	Nanostructured lipid carrier	Artemether	High cumulative permeation rate, high drug stability, high release controllability	In vitro (Caucasian female skin obtained by abdominoplasty)	/	2014	[78]
	Microneedles	Primaquine	Optimal performance, safe for long-term medication	In vitro and in vivo (Wistar rats)	In vitro and ex vivo permeation/release: 31.31 ± 5.25% and 22.55 ± 4.35%.	2021	[79]
Cystic Echinococcosis	Microneedle	Albendazole	High transdermal permeation amount, safe	In vitro and in vivo (rats)	/	2023	[32]
	Microneedles-Nanocrystals system	Albendazole	High transdermal depth, high peak drug concentration, long half-life	Wistar rats	/	2021	[81]
*Lymphatic filariasis*	Solid lipid nanoparticle—microneedle system	Albendazole	High drug targeting efficiency, high direct transport rate, Sustained release, reduced metabolite generation, low drug distribution in liver and kidney	In vitro and in vivo (female Sprague–Dawley rats)	PS: 95.25 ± 9.26 nm; PDI: 0.273 ± 0.02	2019	[83]
*Scabies*, *Rosacea*, *Head lice*, *Trichuriasis*, *Onchocerciasis*, *Lymphatic filariasis*, *Strongyloidiasis*	Microemulsion	Ivermectin	Improved membrane permeability, high solubility	/	PS: 18–54 nm; PDI: 0.3–0.5	2020	[86]
	Nanocrystals	Ivermectin	High equilibrium solubility, fast dissolution rate, high dermal deposition, low side effects	In vitro (Adult pig ear skin)	PS: 186 nm; PDI: 0.4	2023	[87]
	Nanoemulsion (NE)	Ivermectin	Highest drug concentration in stratum corneum and epidermis/dermis junction	In vitro (HaCaT, BJ-5ta, female skin obtained by abdominoplasty)	PS: 57.157 ± 0.455nm; PDI: 0.165 ± 0.014; ZP: −30.600 ± 1.300 mV	2024	[88]
	Nanoemulsion gel (NEG)	Ivermectin	Fastest delivery rate		PS: 106.900 ± 0.490nm; PDI: 0.287 ± 0.037; ZP: −40.400 ± 1.283 mV		
	Colloidal system (CS)	Ivermectin	Highest drug diffusion percentage, efficient delivery		ZP: −36.200 ± 0.666 mV		
	Solid lipid nanoparticles	Ivermectin	Sustained-release efficacy, targeting, low systemic toxicity, few side effects	/	%EE: 99.9±0.0%; %LC: 5.0 ± 0.0%,	2018	[40]
	Nanoparticles	Ivermectin	suitable for acute infection management	/	DL: 10.7%; >90% drug recovery	2024	[89]
	Nanocapsules	Ivermectin	suitable for chronic prevention	/	Z-average: 202 ± 2 nm; ZP: −17 ± 0.5 mV; PDI: 0.12 ± 0.01		
	Hybrid micelles	Eprinomectin	High permeability, low toxicity	In vitro & In vivo (Male Sprague–Dawley rats, Male ICR mice)	PS: 13.97 ± 0.16 nm; PDI: 0.132; %LE: 0.49%; %EE: 95.81%	2024	[90]

Note: “/” indicates that no information was found or is not available. PS: particle size; PDI: polydispersity index; DL: drug loading; EE:entrapped efficiency; HD: Hydrate; ZP: zeta potential.

## Data Availability

No new data were created during the writing of this review article.
